# Single-cell profiling uncovers a rare mesenchymal-like urothelial state associated with sarcomatoid bladder cancer

**DOI:** 10.1038/s41419-026-09136-8

**Published:** 2026-08-04

**Authors:** Justine J. Jacobi, Dongbo Xu, Kyle Wieczorek, Li Wang, Yali Zhang, Jianmin Wang, Prashant K. Singh, Bo Xu, Han Yu, Ahmed A. Hussein, David W. Goodrich, Qiang Li

**Affiliations:** 1 Department of Urology, Roswell Park Comprehensive Cancer Center, Buffalo, NY USA; 2 Department of Biostatistics and Bioinformatics, Roswell Park Comprehensive Cancer Center, Buffalo, NY USA; 3 Department of Cancer Genetics & Genomics, Roswell Park Comprehensive Cancer Center, Buffalo, NY USA; 4 Department of Pathology, Roswell Park Comprehensive Cancer Center, Buffalo, NY USA; 5 Department of Pharmacology and Therapeutics, Roswell Park Comprehensive Cancer Center, Buffalo, NY USA

**Keywords:** Bladder cancer, Oncogenesis, Bladder

## Abstract

The bladder urothelium is a stratified epithelium, composed of basal, intermediate, and umbrella cells. While epithelial–mesenchymal transition is known to occur during bladder regeneration, carcinogenesis, and bladder cancer tumor progression, it remains unclear whether normal urothelial cells intrinsically possess a hybrid mesenchymal-like epithelial state. We analyzed scRNA-seq data of EPCAM+ urothelial cells from both mouse and human urothelium. Here, we identified and characterized a rare population of mesenchymal-like urothelial cells, termed M-cells, representing approximately 1–3% of urothelial cells. Vimentin-positive cells were evaluated by immunostaining to determine their distribution of M-cells in normal urothelial layers. M-cells are enriched within the basal layer but are present across all urothelial compartments. M-cells co-express epithelial markers with mesenchymal and stemness genes and engage stem cell-associated transcriptional regulators. Lineage tracing with the *Upk2-Cre*;*mT/mG* reporter mice confirmed their urothelial origin, excluding a fibroblast lineage. M-cells exhibit high transcriptional plasticity between basal cells and intermediate cells. In addition, we identified a 16-gene transcriptional signature uniquely expressed in M-cells but absent in fibroblasts. RNA deconvolution of MB49 tumors demonstrated strong enrichment of M-cell signature, suggesting that MB49 tumors are more likely to represent sarcomatoid urothelial carcinoma rather than fibroblast-derived sarcoma. The clinical relevance of the M-cell state was validated in primary human sarcomatoid bladder cancer tumors and their corresponding patient-derived xenografts. Together, these findings establish rare and transcriptionally unique M-cells within the normal urothelium as a novel highly plastic population with clinical implications for both mouse and human sarcomatoid urothelial carcinoma.

## Introduction

The urothelium is a highly specialized epithelium that forms a water-tight barrier against urine in the bladder, ureter, and renal pelvis [1]. The urothelium is composed of three distinct layers: basal cells (B cells), which line the basement membrane and function as potential progenitors (marked by KRT5, KRT14); intermediate cells (I cells), which represent a proliferative compartment mediating tissue renewal; and terminally differentiated umbrella (superficial) cells (S cells), which form the luminal barrier (marked by UPK1A/B, UPK2, UPK3A) [1–3]. Recently, single-cell RNA sequencing (scRNA-seq) studies have expanded this classical model by revealing transcriptionally distinct urothelial subtypes and transitional states that underscore the heterogeneity of the bladder urothelium [4–6]. However, the heterogeneity of normal urothelial cells remains incompletely defined, representing a critical gap in understanding how distinct urothelial states contribute to bladder regeneration and tumorigenesis. Moreover, there is a lack of robust animal models that faithfully capture urothelial heterogeneity and enable mechanistic studies of normal urothelial lineage differentiation and tumorigenesis.

Epithelial-mesenchymal transition (EMT) is a conserved process in which epithelial cells lose polarity and adhesion while acquiring mesenchymal traits such as motility, stemness, and transcriptional plasticity [7, 8]. All urothelial cells (B, I, and S cells) can activate EMT-like programs in response to injury, inflammatory cues, or tumor-derived signals [5, 9]. Vimentin (VIM) is a canonical marker of the EMT program and functionally contributes to tissue remodeling, as genetic loss of VIM impairs wound closure and epithelial migration [10, 11]. While EMT activation has been documented during bladder injury, regeneration, and sarcomatoid (SARC) tumor formation, it remains unknown whether normal urothelial cells possess an intrinsic mesenchymal-like epithelial state within the intact uninjured urothelium. Here, we analyzed scRNA-seq data of EPCAM+ urothelial cells from both mouse and human urothelium. We identified a rare population of mesenchymal-like urothelial cells (named as “M-cells”) in both human and mouse urothelium that co-express epithelial markers (EPCAM and KRT8) together with mesenchymal and stemness markers (VIM, AXL, and ZEB1). These M-cells are enriched for EMT- and inflammation-associated programs, display high transcriptional plasticity, and engage distinct transcription factor networks. Moreover, we identified a 16-gene M-cell signature that is absent in fibroblast populations, but enriched in both M-cells and MB49 tumors, suggesting that MB49 tumors are more likely to originate from urothelial M-cells rather than fibroblasts. We further validated the expression of AXL and the therapeutic efficacy of AXL inhibition in MB49 cells, supporting its translational potential for human SARC bladder cancer.

## Materials and methods

### Mouse and human tissue

All animal procedures were approved by the Institutional Animal Care and Use Committee (IACUC) of Roswell Park Comprehensive Cancer Center (RPCCC, protocol #1395). Human studies were conducted in accordance with the Declaration of Helsinki, and written informed consent for the use of remnant bladder tissue in research was obtained from all de-identified patients. Fresh human bladder specimens were collected under Institutional Review Board (IRB) approved protocols (BDR-140921, RPCCC). Fresh human bladder tumor or normal tissues were fixed in 4% paraformaldehyde (PFA) and subsequently embedded in paraffin blocks for histological analysis. MB49 intravesical tumor samples were obtained from a previous study [12]. NSG (NOD.Cg-Prkdc<scid>Il2rg<tm1Wjl > /SzJ) mice of 10 weeks used for patient-derived xenografts were obtained from Roswell Park in-house colony.

### Cell culture and cell viability assays

Mouse cell line MB49 was cultured in DMEM (Corning) with 10% FBS. Cell cultures were maintained at 37 °C in a humidified incubator with 5% CO_2_. The MB49 cell line was obtained from Dr. Ratha Mahendran, Department of Surgery, National University of Singapore [13]. The cell line was routinely tested for mycoplasma contamination. 4000 cells per well were plated in a 96-well plate. Drugs were serially diluted in culture medium and added to the cells. After 72 h, CellTiter-Glo reagent (Promega) was added, and luminescence was measured using BioTek Synergy H1. Drug sensitivity analyses were conducted using GraphPad Prism 10. IC_50_ values were determined by nonlinear regression (curve fitting) with the equation log(inhibitor) versus normalized response (variable slope). Normalized dose–response matrix was analyzed with SynergyFinder Plus [14, 15]. Drug interaction was quantified using four reference models: Bliss independence, Loewe additivity, Highest Single Agent (HSA), and Zero Interaction Potency (ZIP). Mean synergy scores were calculated for each drug pair, and 3D interaction landscapes were generated to visualize synergistic and antagonistic regions within the dose matrix.

### Single-cell RNA library preparation and sequencing

The process of normal urothelial bladder from mouse was described in previous studies [12, 16]. Briefly, male bladder fundus tissues were collected from four *Upk2-Cre*;*mT/mG* mice and then cut into half pieces, and the bladder lumens were unfolded. These tissue pieces were placed in 5.0 mL of biopsy medium containing a 1:10 dilution of collagenase/hyaluronidase (STEMCELL Technologies) and incubated at 37 °C for 1 hour with shaking. Following digestion, the cell pellet was resuspended in 1 mL of TrypLE™ Express Enzyme for 5 minutes, then treated with 1 mL of ACK lysis buffer (Gibco) for 2 minutes. The suspension was filtered using a 40 μm strainer to obtain a single-cell suspension. The alive singlets were isolated via flow cytometry prior to single-cell RNA sequencing.

Single-cell libraries were prepared using the 10X Genomics Chromium Next GEM Single Cell 3ʹ Reagent Kit v3.1. Cell suspensions were first evaluated with Trypan Blue staining on a Countess FL automated cell counter (Thermo Fisher) to assess concentration, viability, and the absence of cell clumps or debris that could interfere with single-cell encapsulation. Cells were then loaded into the Chromium Controller (10X Genomics), where they were partitioned into nanoliter-scale Gel Beads-in-Emulsion (GEMs), each containing a unique barcode for individual cells. Reverse transcription was carried out within the GEMs, followed by amplification of the resulting cDNA. Amplified full-length cDNA was used to construct gene expression libraries through enzymatic fragmentation, end-repair, A-tailing, adapter ligation, and PCR amplification to incorporate Illumina-compatible sequencing adapters. The final libraries were assessed using D1000 screentape on a TapeStation 4200 (Agilent Technologies) and quantified using the KAPA qPCR quantification kit for Illumina platforms (Kapa Biosystems). Libraries were pooled, denatured, and diluted to 300 pM with 1% PhiX control, then loaded onto a NovaSeq Reagent cartridge and sequenced on the NovaSeq 6000 system according to Illumina’s recommended protocol by the RPCCC Genomics Shared Resource.

### Single-cell RNA sequencing processing and analysis

Following sequencing of the mouse 3’ gene expression libraries, raw sequence data were demultiplexed, aligned, and processed for feature calling using the 10x Genomics Cell Ranger pipeline (v8.0.1) using a modified mouse (mm10) genome to include enhanced GFP (“EGFP”) and tdTomato reporter gene sequences. Publicly available human scRNA-seq samples (GSE129845) were mapped to the human (hg38) genome using the 10x Cell Ranger pipeline. Publicly available mouse scRNA-seq fastq files (GSE164557 and GSE240189) were mapped to the mouse (mm10) genome using the 10x Cell Ranger pipeline. High-quality cells that passed standard thresholds (percent mt-reads < 10%; nFeature_RNA > 300 or < 3000; nCount_RNA < 80,000; feature detected in min 3 cells) were further analyzed with Seurat (v4) [17]. Additional doublet detection and filtering were performed with Scrublet [18], and strong exclusivity of broad cell type marker expression was confirmed for each dataset. For datasets with multiple samples, potential batch effects were assessed by visualizing PCA/UMAP embeddings by sample identity, comparing sequencing depth and related QC metrics across samples, evaluating consistency across biological replicates, and examining whether cluster structure and marker gene expression reflected technical source or biological cell state. In the human bladder scRNA-seq dataset from Yu et al. (GSE129845) [19], batch correction was performed using Harmony, consistent with the original study. Gene expression counts were normalized using SCTransform with differences in “percent.mt” regressed out. Following normalization, the top 2000 highly variable features were identified using VariableFeatures. Log-normalized gene expression counts were calculated using Seurat’s NormalizeData() function. Principal component analysis (PCA) was performed to reduce dimensionality, and the first 30–50 principal components (PCs) were selected for downstream clustering and visualization based on elbow plot and variance explained. All cells were initially clustered using the Louvain algorithm with a shared nearest neighbor (SNN) graph (resolution = 0.3). Uniform Manifold Approximation and Projection (UMAP) was used for visualization of the datasets.

Differential expression analysis was conducted using FindAllMarkers from Seurat using MAST (min.pct = 0.25, logfc.threshold = 0.25). Cell annotation mapping on all cells was performed either using Seurat’s MapQuery function using scRNA-seq of published or custom signatures, or using broad annotations from SingleR [20] for mouse and human datasets. Broad cell type annotations were then implemented based on established gene markers from the literature and reference mapping predictions. To identify genes unique in mouse M-cells versus Fibroblasts, differential testing was performed using the FindAllMarkers function in Seurat. We then overlapped all significant differentially expressed genes from each cell type. Shared genes were expressed in both cell types, while genes that were only found in each of the cell types were considered unique. This generated the 16-gene M-cell unique signature. Otherwise, an M-cell signature consisting of the top 50 most significantly upregulated genes in M-cells (vs other urothelial cells) was used and indicated in the text. When queried on human datasets, the 50-gene M-cell signature was mapped to human orthologs (>93% mapping). Pathway analysis of Hallmark gene sets (mouse) was performed using SeuratExtend [21] via AUCell. Custom module scoring was performed with AUCell/GSVA [22].

To quantify cellular plasticity at single-cell resolution, we applied CytoTRACE2 [23, 24], a deep learning-based computational tool that infers differentiation potential from scRNA-seq data and visualized these scores per cell, grouped by bladder urothelial cell type. Transcription factor regulatory networks were determined at single-cell resolution with SCENIC [25] and grouped by urothelial cell type. Pearson’s correlation was used to assess the correlation between gene expression and signature scores, per cell. To visualize the EGFP+ Upk2-driven mouse urothelial cells, the 2,123 EPCAM+ urothelial cells were filtered for cells with counts of EGFP > 1 using Seurat’s subset() function and reprojected in UMAP space, yielding 711 EGFP+ urothelial cells.

### Bulk RNA sequencing analysis and deconvolution

The RNA extraction, library preparation, and sequencing methods from mouse normal and MB49 tumor tissues are previously described [12]. Bulk RNA-seq reads were first assessed for quality using fastqc and then mapped to the mouse GRCm38-mm10 genome using STAR and quantified with RSEM [26, 27]. Features were filtered if not expressed in more than 3 samples. Normalized counts were calculated within DESeq2 [28] with variance stabilization. Bulk RNA-seq samples were deconvoluted using BayesPrism [29] with the single-cell reference derived from normal mouse bladder scRNA-seq. Transcriptomic count matrices were used as input for both bulk and single-cell data. Genes expressed in fewer than 10 cells were excluded from the reference, and cell types with fewer than 50 cells were removed prior to model fitting. BayesPrism was run with default priors and 10,000 Gibbs sampling iterations, and posterior estimates of cell-type fractions and cell type–specific gene expression were used for visualization.

### Immunohistochemistry and immunofluorescence

Sections (5.0 μm) of paraformaldehyde-fixed and paraffin-embedded mouse or human normal bladder specimens were prepared for immunostaining. Primary antibodies used included Vimentin (Cell Signaling Technology, #5741), KRT8 (DSHB, #TROMA-I-s), and GFP (Santa Cruz, #sc-9996), UPK3 (Fitzgerald, #10R-U103A), KRT5 (BioLegend, #905901), and AXL (Cell Signaling Technology, #18554S), CD44 (Invitrogen, #14-0441-82), E-cadherin (Cell Signaling Technology, #3195S), ZEB1 (Cell Signaling Technology, #70512S), FN1 (Cell Signaling Technology, #26836S) and Vimentin (Invitrogen, #MA5-11883). Immunohistochemistry (IHC) was performed using the VECTASTAIN Elite ABC kit and DAB substrate (Vector Labs), following the manufacturer’s and reported protocol [12, 30]. For IHC-immunofluorescence (IHC-IF), sections were incubated with primary antibodies, followed by a 1-hour incubation with Alexa Fluor 594- or 488-conjugated secondary antibodies, and then mounted using mounting medium with DAPI.

### Clinical associations using publicly available data

TCGA-BLCA RNA-seq and clinical annotations were obtained from TCGA, and GSE87304 from Seiler et al [31] microarray data and metadata were obtained from GEO. For each sample, an M-cell signature score was calculated as the mean of gene-wise Z-scored expression values across the genes in the human M-cell signature. In TCGA-BLCA, overall survival was evaluated by Kaplan-Meier and Cox proportional hazards analyses.

### Statistical analysis

All bioinformatic and public-cohort analyses were performed in R (version 4.2.0) using the indicated packages. Pearson’s correlation was used to assess correlations between gene expression and signature scores on a per-cell basis. A two-sided t-test was used to test for non-zero Pearson’s correlation coefficient. For TCGA-BLCA, overall survival was evaluated by Kaplan-Meier analysis, with differences between groups assessed using the log-rank test. For GSE87304, differences in M-cell signature scores across molecular subtype groups were assessed using the Kruskal-Wallis test. The percentages of VIM+ epithelial cells were represented as median and inter-quartile range, and compared between male and female mice/human using Wilcoxon rank-sum test. Data points within each subject were averaged and treated as a single observation for hypothesis testing. Co-IF of KRT8, GFP, KRT5, and UPK3 cells percentages data were represented as mean ± SD for descriptive purposes, as median values were 0 for several variables due to a floor effect/high proportion of zero values, making the median insufficiently informative regarding the distribution and magnitude of the observed data. All tests were two-sided and conducted at a significance level of 0.05.

## Results

### Single-cell profiling uncovers Vimentin-positive M-cells in normal bladder urothelium

**Fig. 1:**
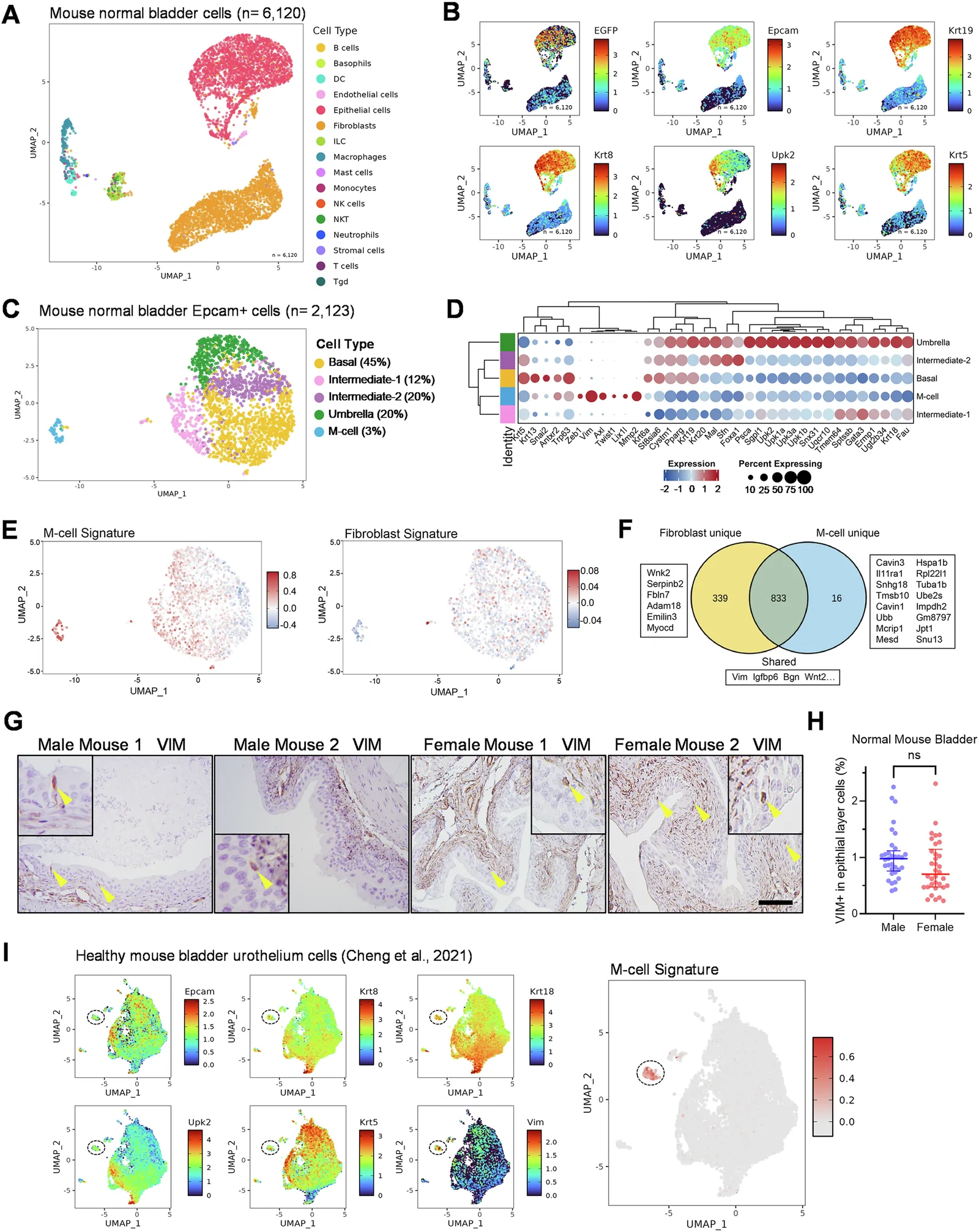
Single-cell profiling uncovers Vimentin-positive mesenchymal-like cells in normal mouse bladder. **A** UMAP projection of 6,120 single cell transcriptomes (scRNA-seq) derived from mouse normal bladder tissue colored by broad cell type classification. **B** UMAPs of cells colored by normalized expression of select urothelial cell marker genes. **C** Cells from (**A**) were isolated based on Epcam, Krt18, and Krt19 positive expression and re-projected in UMAP space with cells colored by urothelium cell type. Percentages indicate proportion of cells within urothelial (Epcam + /Krt18 + /Krt19 + ) population. **D** Hierarchical clustering of urothelial cell types (Identity) based on differential gene expression markers for each cell type. Dot color indicates scaled gene expression and size indicates percentage of cells within each cell type expressing each gene. **E** Signature scoring (GSVA) of gene sets unique to M-cells (16-gene) “M-cell Signature” or genes unique to Fibroblasts “Fibroblast Signature” per cell and visualized within the UMAP projection. GSVA color scale indicates positive (red) or negative (blue) gene set activity. **F** Venn diagram showing the unique and shared genes across mouse fibroblast and M-cell urothelial cells. Select genes are highlighted and detailed in Table S3. **G** Representative immunohistochemistry images with vimentin staining in mouse male and female bladder tissues. Scale bar: 100 μM. Yellow arrow: positive staining cells. **H** Quantification of vimentin positive staining cells located in mouse male and female urothelium. ns: not significant (*p* = 0.07). Each dot represents data obtained from a single imaging field. Multiple fields were collected from normal bladder tissues of 9 male and 7 female mice. Data are presented as the median with interquartile range. **I** Analysis of healthy mouse urothelium scRNA-seq dataset (GSE164557) from Cheng et al. [5], showing UMAP projections of urothelial cells co-expressing urothelial markers and Vimentin (Vim). Dashed circle indicates analogous M-cell population (left) identified in this dataset. 16-gene “M-cell Signature” enrichment within bladder urothelial cells (right). GSVA color scale indicates positive (red) or negative (blue) gene set activity.

To investigate intrinsic urothelial heterogeneity and plasticity, we performed scRNA-seq on urothelial cells from four normal mouse bladders. Reference-based annotations revealed predominantly epithelial (43.6%) and fibroblast (45.2%) populations, with limited immune infiltration (10.2%) (Fig. 1A). Epithelial cells expressed canonical markers Epcam, Krt18, Krt8, and Krt19 (Fig. 1B; Table S1). Using Epcam, Krt18, and Krt19 expression, we identified approximately 2100 urothelial cells with high epithelial marker expression. Within this subset, distinct cell types were defined by top differentially expressed genes: Basal (Trp63, Krt5), Umbrella (Upk2, Upk3a, Krt20, Gata3), and two Intermediate populations (Gata3-/Krt5 + /Foxa1 + /Krt20+ and Gata3 + /Krt5-/Foxa1-/Krt20-) (Fig. 1C, D; Table S2).

A small subset (3% of epithelial cells) expressed Epcam but low Trp63 and elevated mesenchymal markers (Vim, Zeb1, Twist1, Mmp2), designated as “M-cells.” Comparison with fibroblasts (Table S3) showed overlap in genes such as Vim, yet M-cells exhibited strong activation of a distinct 16-gene signature enriched in this population relative to fibroblast-specific genes (Fig. 1E, F). Notably, low-level expression of these M-cell genes was also detected in subsets of other urothelial cells, suggesting partial activation of the M-cell program. To validate this, we performed IHC staining for Vimentin on normal bladder tissues from male and female mice, revealing rare Vimentin-positive cells within the urothelium, more enriched in the basal layer (Fig. 1G). Quantification showed about 0.98% Vimentin-positive urothelial cells in male bladders and 0.70% in females, with no significant sex difference (*p* = 0.07, median value, Fig. 1H). To validate M-cells in an independent scRNA-seq dataset by Cheng et al. [5], we confirmed a small M-cell population (Vim + /Epcam + , with mixed Krt8+ and Krt5 + ) exhibiting the same 16-gene M-cell signature in healthy mouse bladder urothelium (Fig. 1I). These results indicate that M-cells express epithelial markers such as Epcam, Krt8, Krt18, and Krt19, suggesting their urothelial origin rather than mesenchymal origin. The presence of M-cells suggests an intrinsic epithelial-mesenchymal transition or a plastic hybrid state within the normal urothelium. Together, these findings demonstrate that the normal bladder urothelium contains a rare population of transcriptionally distinct mesenchymal-like urothelial cells (M-cells) with a unique 16-gene signature absent in fibroblasts.

### Lineage labeling with Uroplakin-2 confirms that M-cells arise from the urothelium

**Fig. 2:**
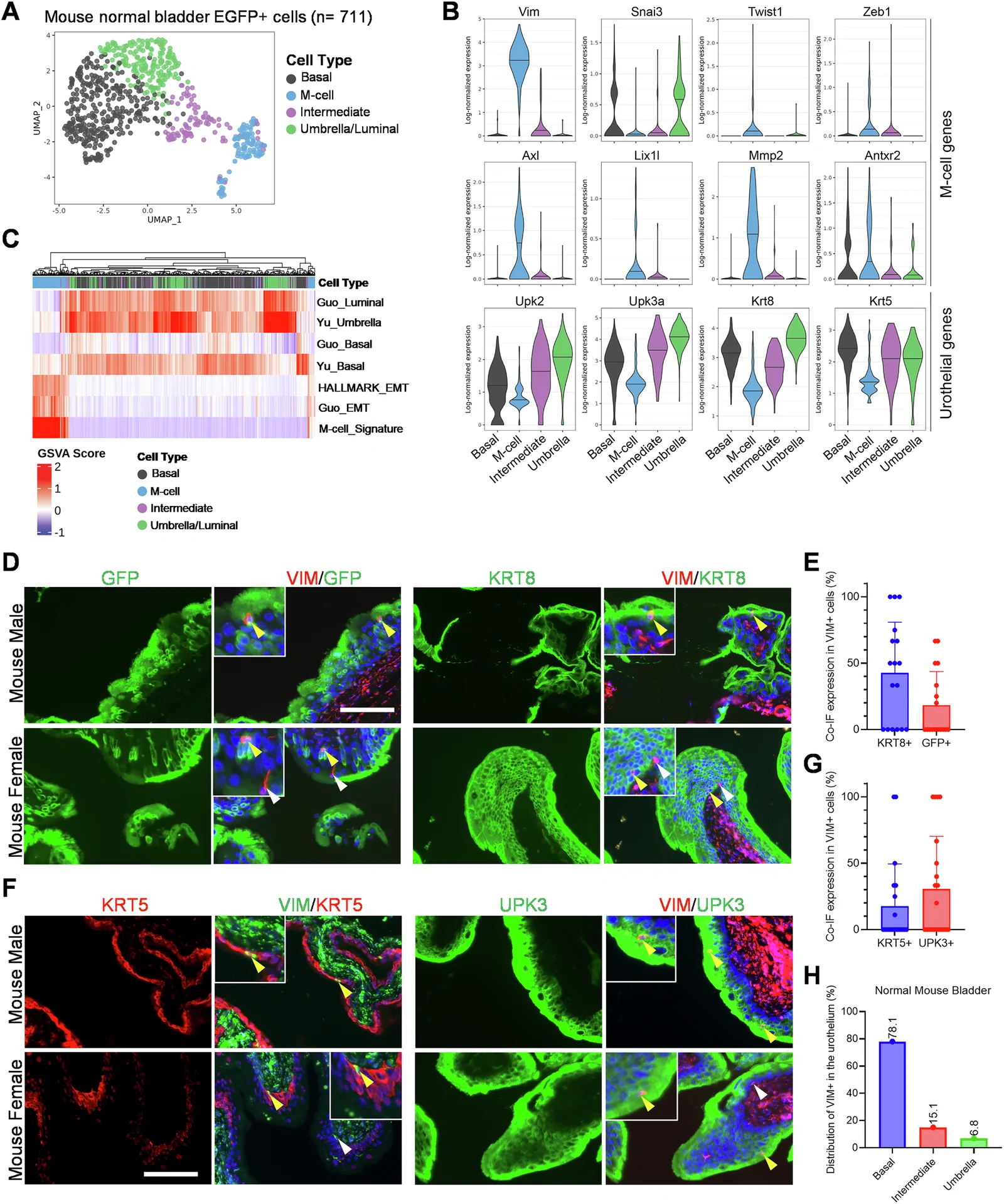
Uroplakin-2 lineage labeling confirms M-cells are a rare mesenchymal-like population of urothelial origin. **A** Isolated urothelial cells (*n* = 711) with positivity for EGFP+ reporter expression from Fig. 1A with cells colored by urothelium cell types. **B** Violin plots of M-cell (top two rows) and urothelial (bottom row) marker normalized gene expression per cell and grouped by cell type within the EGFP+ population. **C** Signature scoring (GSVA) of curated gene sets from Guo et al [33] and Yu et al.[19] per cell and visualized as a scaled heatmap. Top color annotation bar indicates EGFP+ urothelial cell type annotation from (**A**). GSVA color scale indicates positive (red) or negative (blue) gene set activity. M-cell signature consists of the top 50 most significantly upregulated genes in M-cell cell type (vs rest) from Fig. 1. **D** Co-immunofluorescence staining of vimentin and GFP or KRT8 in mouse male and female bladder tissues. Scale bar: 100 μM. Yellow arrow: colocalization; White arrow: non-colocalization. **E** Percentage of KRT8 or GFP co-expressing cells in vimentin (red) positive cells located in mouse urothelium. Each dot represents data obtained from a single imaging field. Multiple fields were collected from normal bladder tissues of 3 male and 3 female *Upk2-Cre;mT/mG* mice. Data are presented as mean ± SD. **F** Co-immunofluorescence staining of vimentin and KRT5 or UPK3 in mouse male and female bladder tissues. Scale bar: 100 μM. Yellow arrow: colocalization; White arrow: non-colocalization. **G** Percentage of KRT5 or UPK3 co-expressing cells in vimentin positive cells located in mouse urothelium. Each dot represents data obtained from a single imaging field. Multiple fields were collected from normal bladder tissues of 3 male and 3 female *Upk2-Cre;mT/mG* mice. Data are presented as mean ± SD. **H** Distribution of vimentin positive cell in mouse different urothelial layers (basal, intermediate and umbrella). Data were calculated based on the total number of VIM+ cells across imaging fields from bladder tissues of 9 male and 7 female mice.

To confirm the urothelial lineage of M-cells, we analyzed the epithelial subset of our normal mouse bladder scRNA-seq dataset (Fig. 1) for cells expressing the EGFP reporter (Fig. 1B) driven by the urothelium-specific *Upk2* promoter in *Upk2*-Cre;*mT/mG* mice [32]. In adult urothelium, EGFP marks cells with a history of UPK2 expression (urothelial origin). This yielded approximately 711 EGFP+ cells spanning basal, intermediate, and umbrella populations (Fig. 2A). Within this subset, M-cells formed a minor population enriched for mesenchymal markers (Vim, Twist1, Zeb1, Mmp2, Axl), previously identified in EMT-enriched SARC tumors by Guo et al. [33] (Fig. 2B). From the broader EPCAM+ epithelial dataset, we also derived a 50-gene M-cell signature representing the top upregulated genes in M-cells versus other urothelial cells. The EGFP + M-cell subset showed strong enrichment for this signature as well as HALLMARK-EMT and Guo-EMT programs (Fig. 2C).

To validate co-expression of VIM with urothelial markers, we performed immunofluorescence staining for VIM, GFP, and KRT8 in *Upk2*-Cre;*mT/mG* male and female bladder tissue. VIM colocalized with both GFP and KRT8 in subsets of urothelial cells (Fig. 2D), although some VIM+ cells lacked GFP or KRT8. Quantification showed 42.6% of VIM+ cells expressed KRT8, and 18.3% expressed GFP, indicating Upk2 lineage (Fig. 2E). Additional staining for KRT5 and UPK3 revealed 17.6% and 30.6% of VIM+ cells positive for these markers, respectively (Fig. 2F, G). Spatially, most VIM+ cells (78.1%) were distributed in basal layers, with fewer in intermediate (15.1%) and umbrella (6.8%) layers (Fig. 2H). Together, lineage tracing with the *Upk2*-Cre;*mT/mG* model confirms that a subset of VIM + M-cells arises from the urothelium and partially co-expresses urothelial markers, including KRT8, KRT5, and UPK3.

### EMT-like and plastic transcriptional programs define M-cell population

**Fig. 3:**
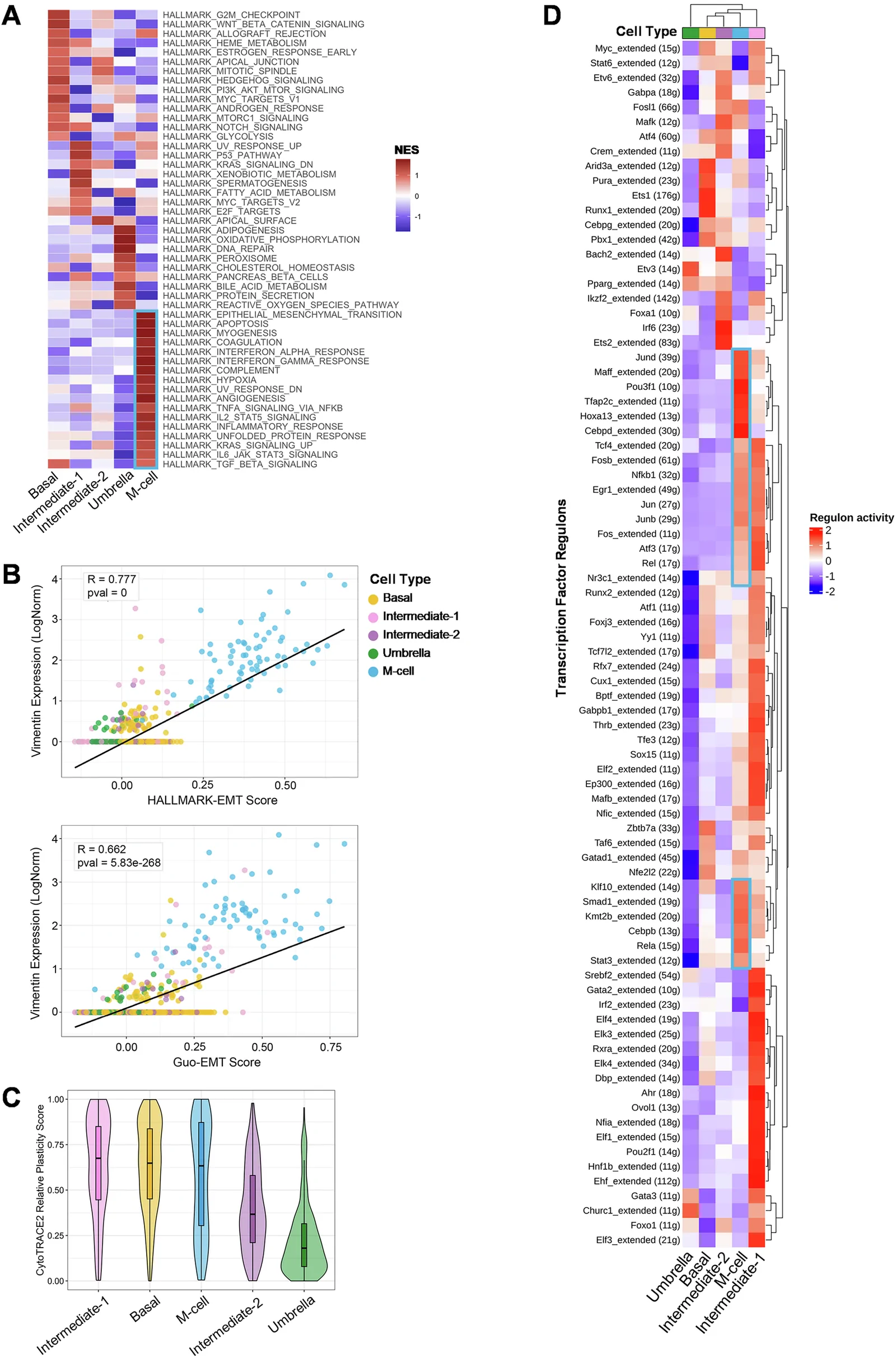
EMT-like and plastic transcriptional programs define the M-cell population. **A** Gene set enrichment analysis (GSEA) on gene expression from subsetted urothelial (Epcam + /Krt18 + /Krt19 + ) cells from Fig. 1C using Hallmark mouse pathways from MSigDb. Cells were aggregated across cell type, and scores are presented as Normalized Enrichment Scores (NES). Pathways enriched in M-cells are indicated with blue boxes. **B** Scatter plots correlating Vimentin (Vim) gene expression with MSigDb Hallmark EMT pathway or mesenchymal genes from Guo et al.’s EMT pathway [33]. Each point represents a cell, colored by cell type from Fig. 1C. Correlation coefficients (R) and corresponding *p*-values were calculated using the Pearson correlation test. **C** Relative CytoTRACE2 transcriptional plasticity scores per cell and grouped by urothelial cell type as described in Fig. 1C. Relative plasticity scores range from 1 (less differentiated, more plastic) to 0 (more differentiated, less plastic). Horizontal line indicates median score per cell type. **D** Heatmap showing transcription factor regulon activity scores per cell type calculated by SCENIC. Top enriched transcriptional regulators are shown. The number of genes within each transcription factor regulon is inside parentheses. Regulators enriched in M-cells are indicated with blue boxes. Positive transcription factor regulon activity is indicated in red on the color scale, and blue indicates negative regulon activity.

To determine whether M-cells exhibit a mesenchymal transcriptional phenotype, we performed gene set enrichment using the mouse HALLMARK collection. M-cells were enriched for EMT-like and inflammatory programs, including EPITHELIAL MESENCHYMAL TRANSITION (EMT), TGF-beta SIGNALING, and INFLAMMATORY RESPONSE (Fig. 3A). As Vimentin marks EMT and stem-like states, we assessed whether its expression correlated with EMT activity. Indeed, increasing Vim expression strongly correlated with high HALLMARK-EMT scores (Fig. 3B). As additional validation, we applied the EMT gene set from Guo et al. [33], previously used to define mesenchymal-like sarcomatoid bladder tumors enriched for TGF-beta and Rho signaling but depleted of epithelial markers. M-cells with higher Vim similarly showed stronger Guo-EMT signature activity.

To determine whether M-cells exhibit a transcriptionally permissive phenotype, we applied CytoTRACE2 [23, 24], a scRNA-seq algorithm that infers cellular potency from gene-expression diversity, with higher scores reflecting less differentiated and more plastic cellular states. Umbrella cells scored lowest, consistent with terminal differentiation, whereas M-cells displayed broadly elevated plasticity scores comparable to intermediate and basal urothelial cells, suggesting that M-cells retain a less differentiated, transcriptionally plastic state (Fig. 3C). To further characterize the regulatory programs underlying the M-cell transcriptional phenotype, we used SCENIC [25] to infer cell type-specific transcription factor regulons based on coordinated expression of transcription factors and their predicted target genes (Fig. 3D). Canonical umbrella, basal, and intermediate cells showed Gata3, Pparg, and Foxa1 activity, whereas M-cells exhibited distinct activation of Hoxa13, Tfap2c, and Pou3f1—factors implicated in EMT during development and cancer [34–36]. M-cells and intermediate cells also shared enrichment for inflammatory regulators, including Nfkb1, Smad1, and Jun/Fos family members [37, 38]. Together, these findings describe a rare, potentially plastic M-cell population within the normal bladder urothelium characterized by mesenchymal and inflammatory transcriptional programs.

### Vimentin-positive M-cells exist in bladder urothelium from healthy human donors

**Fig. 4:**
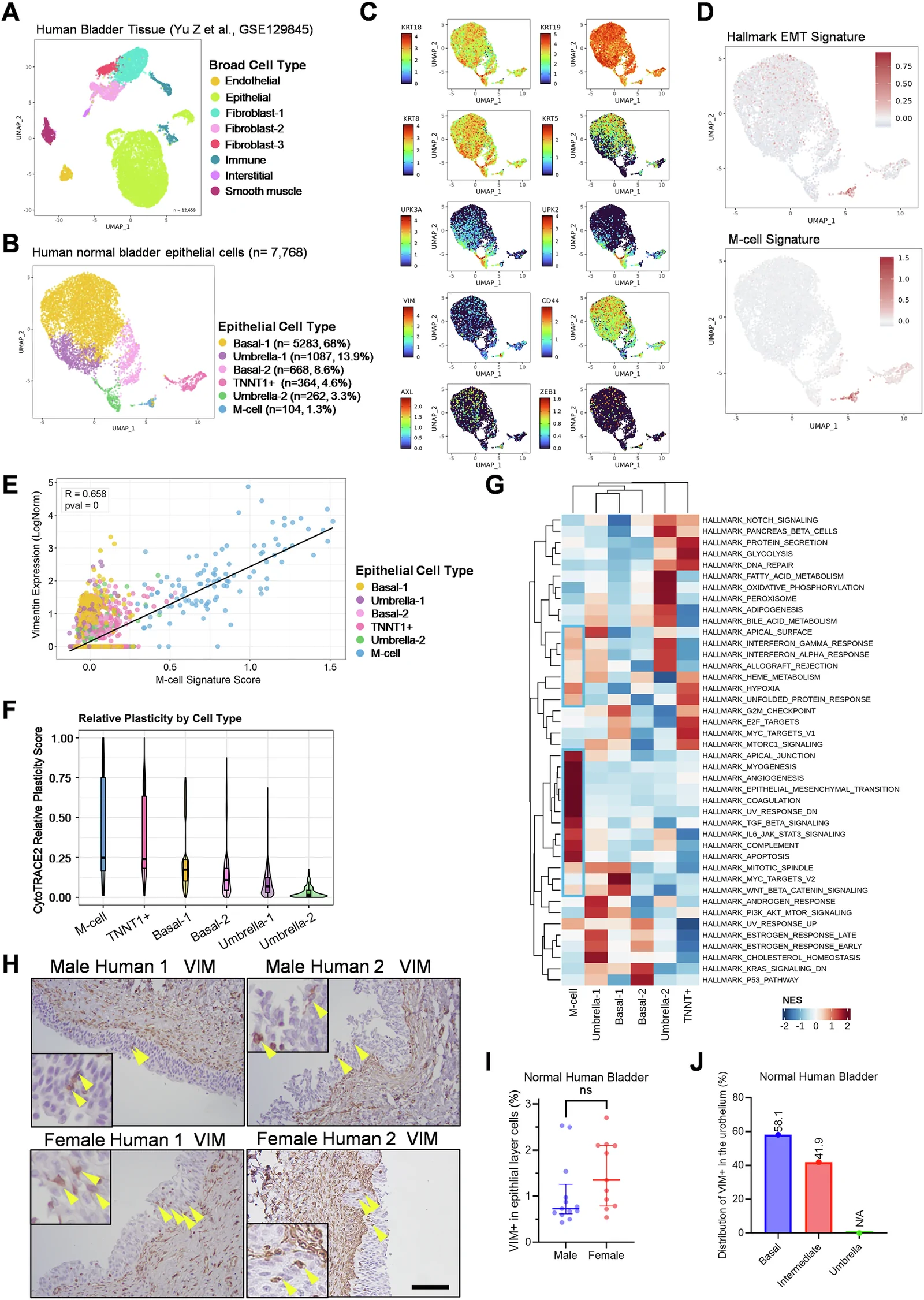
VIM + M-cells exist in bladder urothelium from healthy human donors. **A** UMAP projection of 12,659 individual transcriptomes of published human healthy bladder donor samples from Yu et al. (GSE129845) [19]. Each cell colored by broad cell type annotated using SingleR human cell type reference. **B** Isolated urothelial (EPCAM + ) cells from (**A**) re-projected in UMAP space and colored by cell type. Legend indicates the number of cells and percentage of total for each urothelial cell type. **C** UMAPs of EPCAM+ cells colored by epithelial cell marker expression and M-cell type marker genes (VIM, AXL, ZEB1). **D** UMAP of EPCAM+ urothelial cells colored by gene signature scoring (GSVA) of MSigDb Human Hallmark EMT signature or our broader 50-gene M-cell signature derived from M-cells in Figs. 1 and 2. Color scale indicates positive (red) or negative (blue) gene set activity within each cell. **E** Scatter plots correlating Vimentin (VIM) gene expression with M-cell signature activity. Each point represents an EPCAM+ cell, colored by annotated cell type from (**B**). Correlation coefficients (R) and corresponding *p*-values were calculated using the Pearson correlation test. **F** Relative CytoTRACE2 transcriptional plasticity scores per EPCAM+ cell and grouped by human urothelial cell type. Relative plasticity scores range from 1 (less differentiated, more plastic) to 0 (more differentiated, less plastic). **G** GSEA on subsetted urothelial cells using MSigDb Human Hallmark gene pathways. Cells were aggregated across cell types, and scores are presented as NES with hierarchical clustering between annotated EPCAM+ cell types. Pathways enriched in M-cells are indicated with blue boxes. **H** Representative immunohistochemistry images with vimentin staining in human male and female bladder tissues. Scale bar: 100 μM. Yellow arrow: positive staining cells. **I** Quantification of vimentin positive staining cells located in human urothelium. ns: not significant (*p* = 0.32). Each dot represents data obtained from a single imaging field. Multiple fields were collected from normal bladder tissues of 7 male and 7 female human donors. Data are presented as the median with interquartile range. **J** Distribution of vimentin positive cell in human different urothelial layers (basal, intermediate and umbrella). Data were calculated based on the total number of VIM+ cells across imaging fields from bladder tissues of 7 male and 7 female human donors.

Next, we investigated whether a mesenchymal-like M-cell ortholog exists in healthy human urothelium. We re-analyzed the scRNA-seq dataset from Yu et al [19], comprising over 12,000 cells from healthy donors, including epithelial, stromal, and immune populations (Fig. 4A). Using SingleR-based reference mapping, we identified 7768 epithelial cells expressing canonical epithelial markers across six transcriptionally distinct clusters, including a glycolytic-TNNT1+ epithelial population reported by Yu et al. (Fig. 4B, C) [19]. To determine whether a human population analogous to mouse M-cells exists, we performed per-cell signature scoring (GSVA) using the HALLMARK-EMT gene set and a 50-gene M-cell signature mapped to human orthologs. This revealed a distinct epithelial cluster with strong enrichment of both transcriptional programs (Fig. 4D), positive correlation with VIM expression, and the highest transcriptional plasticity among urothelial clusters (Fig. 4E, F). Like mouse M-cells, human M-cells were enriched for EMT and regenerative signaling pathways, including TGF-beta and IL6-JAK/STAT (Fig. 4G).

To validate histologically, we performed Vimentin IHC on independent normal human bladder tissue. Consistent with mouse findings, only rare VIM+ cells were detected in both male (0.73%, median value) and female (1.35%, median value) urothelial layers (Fig. 4H, I). The VIM+ cells were distributed in basal (58.1%) and intermediate (41.9%) layers (Fig. 4J). The umbrella layer was often absent in the surgical specimens, likely due to surgical or mechanical disruption during tissue procurement and preparation. VIM+ cell frequency did not differ significantly between sexes (*p* = 0.32). We further examined the expression of additional EMT biomarkers, including AXL and E-cadherin (E-cad), in normal human bladder tissue. Partial colocalizations of AXL, E-cad, along with the stem cell-like marker (CD44), were observed in M-cells (VIM marker) from both male and female donors (Fig. S1). Collectively, these data confirm the presence of rare VIM + M-cells in normal human bladder urothelium, enriched for EMT, regenerative pathways, and stem cell markers, primarily localized to the basal layer.

### Transcriptional programs of urothelial M-cells are recapitulated in MB49 tumors

**Fig. 5:**
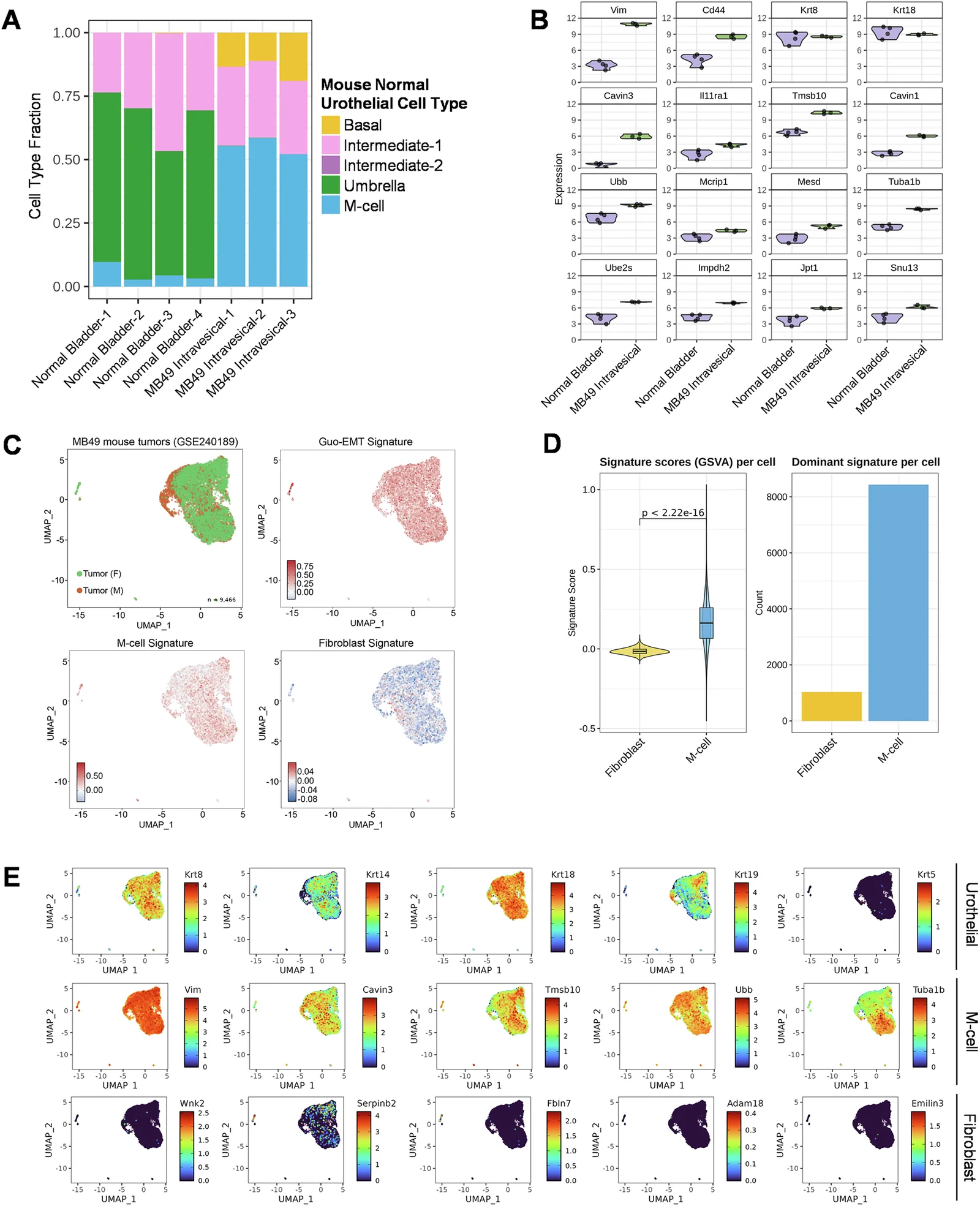
Transcriptional programs of M-cells are recapitulated in mouse sarcomatoid bladder tumors. **A** RNA deconvolution using BayesPrism of bulk transcriptomes from normal (Normal Bladder 1–4) tissue or sarcomatoid bladder cancer mouse models (MB49-Intravesical 1-3). Normal bladder urothelial cell types from Fig. 1C were used as the scRNA-seq input for deconvolution. Y-axis indicates the cell type fraction predicted within queried samples (X-axis) based on scRNA-seq cell types (Mouse Normal Urothelial Cell Type). **B** Log-transformed gene expression (log-TPM) of either M-cell markers (Vim and Cd44), urothelial markers (Krt8/18) or the twelve of the sixteen genes found to be uniquely expressed in mouse M-cells compared to mouse fibroblasts from scRNA-seq analysis. Bulk RNA-seq counts of each gene are plotted per sample and grouped based on mouse model. **C** UMAPs of published MB49 mouse tumors (GSE240189) from male (*n* = 1) and female (*n* = 1) tumor cells (total *n* = 9466). UMAPs of cells colored either by tumors derived from male or female mouse tumors or by signature score activity. MB49 cells colored by gene signature scoring of EMT signature from Guo et al. [33], or the unique 16-gene unique M-cell signature, or Fibroblast unique gene signature derived from cells in Figs. 1 and 2. Color scale indicates positive (red) or negative (blue) gene set activity. **D** Quantification of M-cell and Fibroblast unique signature scores in MB49 tumors, calculated per cell from (**C**) using GSVA. Left, per-cell signature scores are shown as violin plots with overlaid mean/inter-quartile range; *p*-value was calculated using Welch’s t-test. Right, bar plot showing the dominant signature per cell, defined as the signature with the higher enrichment score in each cell. **E** UMAPs showing normalized gene expression per cell of either urothelial, M-cell unique, or Fibroblast-unique genes within MB49 tumor cells from (**C**).

The MB49 cell line, derived from a chemically induced bladder carcinoma in C57 BL/6 mice, has been widely used for over four decades to study tumor biology, therapeutic response, and immune mechanisms in urothelial carcinoma [39, 40]. However, transcriptional profiling shows MB49 cells and tumors more closely resemble fibroblasts [41]. To test whether the rare Vimentin-positive mesenchymal-like population may represent a potential urothelial origin of MB49, we deconvoluted bulk RNA-seq profiles from normal mouse bladder tissue and MB49 intravesical tumors using our urothelial scRNA-seq cell state signatures (Fig. 1C). Given that rare epithelial states are difficult to resolve from bulk RNA-seq alone, deconvolution allowed us to assess whether MB49 tumors preferentially recapitulate transcriptional programs associated with defined normal urothelial populations. Deconvolution predicted normal bladder samples were dominated by Umbrella and Intermediate-1 cell types, with <10% M-cells (Fig. 5A), whereas MB49 tumors showed >50% enrichment for M-cells along with Basal and Intermediate-1 populations. Given the fibroblast-like characteristics of MB49 tumors, we next examined expression of the 16 genes uniquely upregulated in M-cells relative to fibroblasts (Table S1). All were expressed in MB49 tumors (Fig. 5B).

To validate these similarities between M-cells and MB49 tumor cells, we re-analyzed publicly available scRNA-seq data from MB49 tumors [42] composed of CD45-negative tumor cells from male and female mice (Fig. 5C). These cells were enriched for the Guo-EMT signature, as expected, and showed strong positive enrichment for the 16-gene M-cell signature but negative enrichment for the fibroblast-specific signature (Fig. 5C, D). MB49 cells co-expressed urothelial and M-cell genes, with minimal/no fibroblast-specific expression (Fig. 5E). Together, the transcriptional similarity between MB49 tumors and normal M-cells, along with the absence of a fibroblast signature, suggests that MB49 tumors are more likely derived from a urothelial lineage, possibly M-cells, rather than a fibroblast lineage.

### MB49 tumors exhibit urothelial M-cells features and sensitivity to AXL Inhibition

**Fig. 6:**
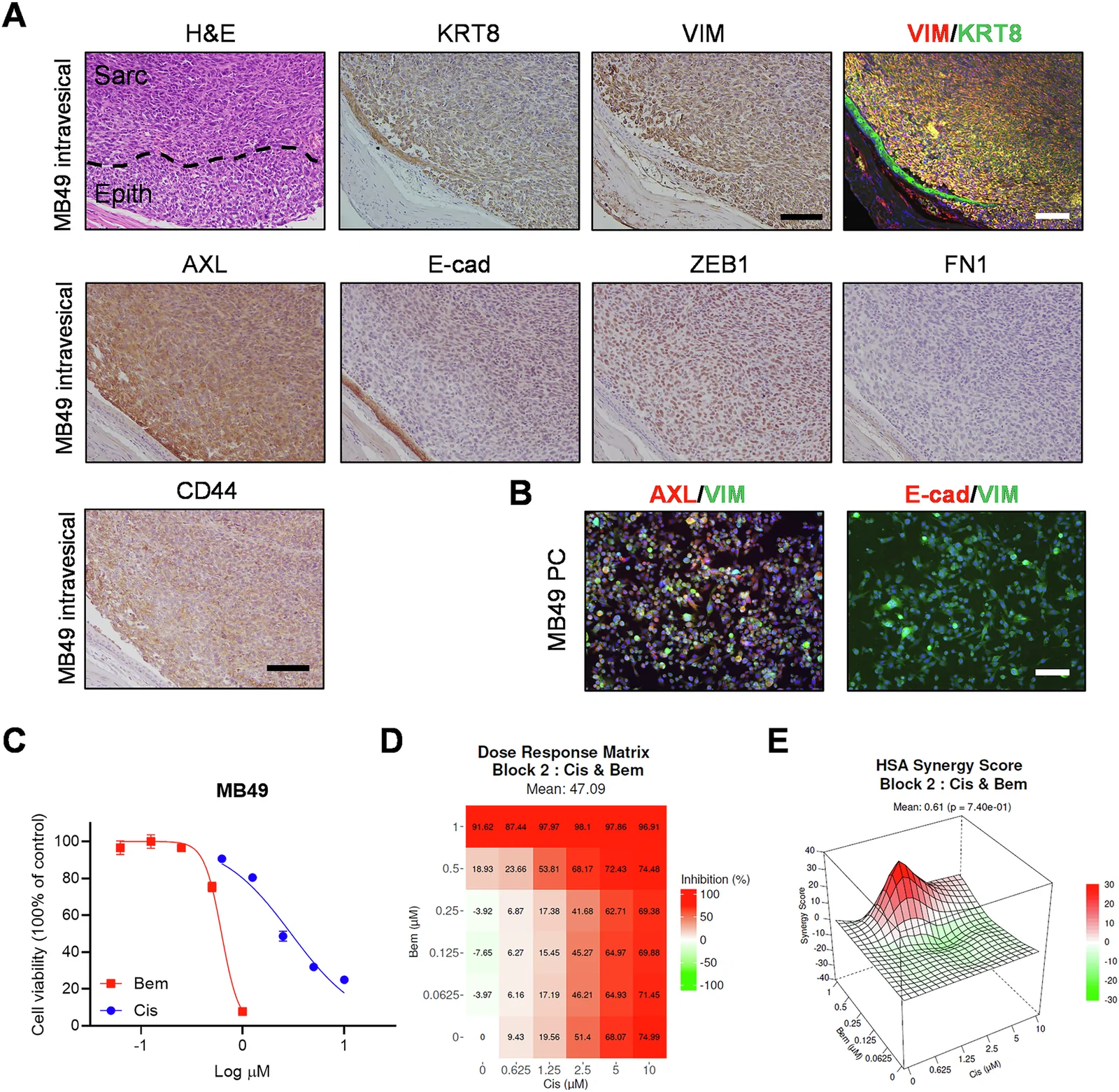
MB49 tumors exhibit urothelial M-cells features and sensitivity to AXL Inhibition. **A** H&E and IHC staining with KRT8, VIM, AXL, E-cad, ZEB1, FN1 and CD44 in MB49 tumors from C57 BL/6 intravesical model tumors. Sarc: sarcomatoid histology; Epith: epithelial histology. Scale bar: 100 uM. **B** Co-immunofluorescence staining of vimentin and AXL or E-cad in MB49 cells in cell culture dish. **C** Single drug response of Cisplatin (Cis) or AXL inhibitor Bemcentinib (Bem) in MB49 cells. Cell viability was measured with CellTiter Glo after 72 h treatment. Visualization of the inhibition response matrix (**D**) and the synergy score 3D plot (**E**) in MB49 cells with co-treatment using HSA drug interaction model by SynergyFinder Plus.

To validate the bioinformatic findings in MB49 tumor, we performed IHC analysis of EMT and stem cell-like biomarkers in the MB49 intravesical tumor model established in C57 BL/6 mice. Histology confirmed co-expression of KRT8 and VIM in MB49 tumors (Fig. 6A). Also, MB49 intravesical tumor clearly exhibits two distinct populations of urothelial and sarcomatoid histology. Strong expression of the urothelial marker KRT8, EMT markers (VIM, AXL, and ZEB1), and the stem cell-like marker CD44 was confirmed in MB49 tumors (Fig. 6A).

Next, we also confirmed the strong expression of AXL and VIM in MB49 cells (Fig. 6B). To investigate the potential clinical application of the AXL inhibitor bemcentinib (Bem) in MB49 cells, we investigated the single drug response and co-treatment of Bem and Cisplatin (Cis). AXL inhibitor Bem (IC_50_ = 0.62 µM) exhibited better efficacy comparable to that of cisplatin (IC_50_ = 3.0 µM) in MB49 cells (Fig. 6C), and co-treatment of Bem (0.5 µM) and Cis (1.25 µM) exhibited the moderate synergistic effect calculated by HSA drug interaction model with SynergyFinder Plus (Fig. 6D, E). Together, these findings suggest that AXL inhibition with bemcentinib may represent a promising strategy for sarcomatoid bladder cancer.

### M-cell transcriptional program associates with aggressive disease in human bladder cancer cohorts

**Fig. 7:**
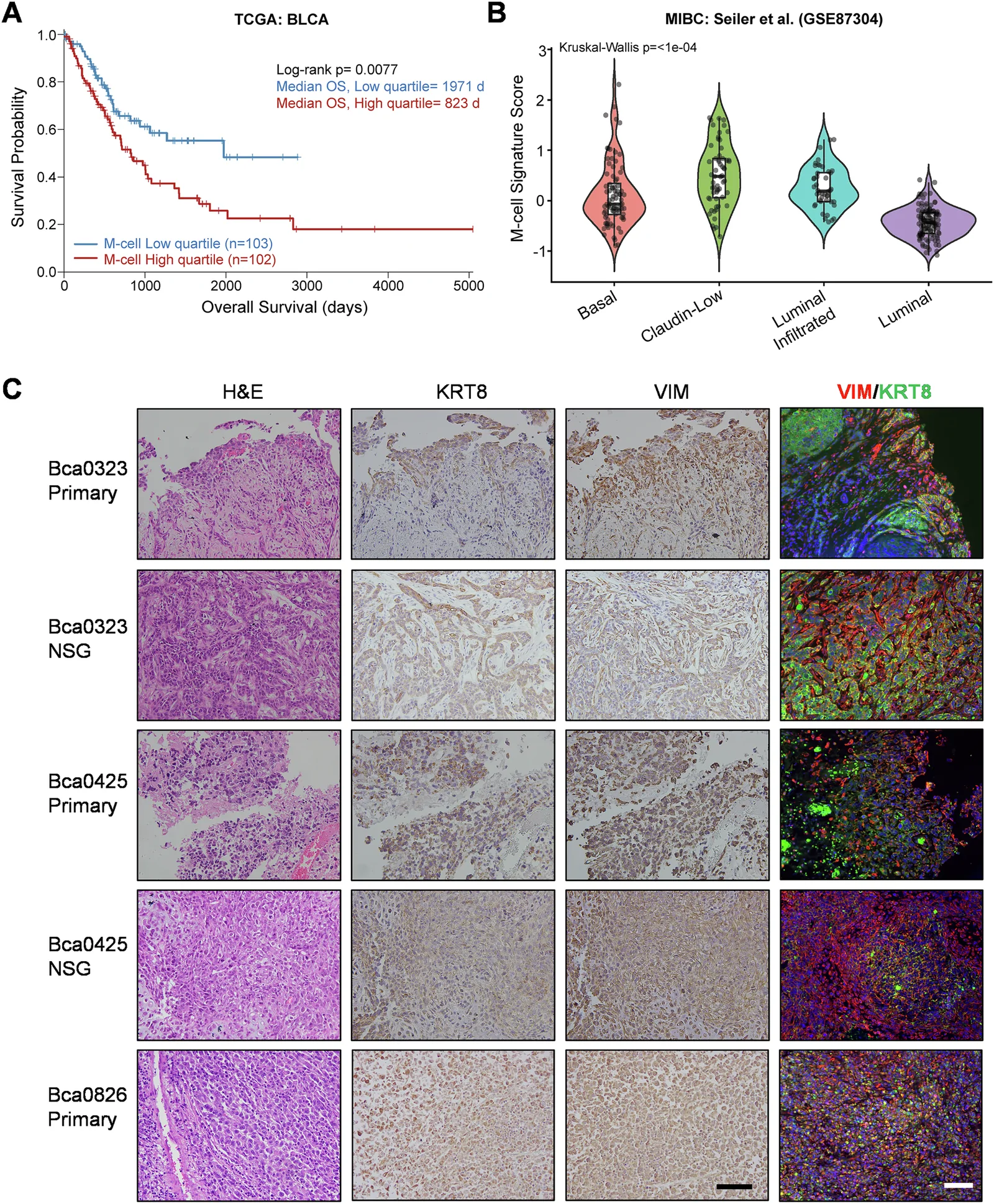
M-cell transcriptional program associates with more aggressive disease in human bladder cancer cohorts. **A** Kaplan-Meier analysis of overall survival in TCGA-BLCA primary tumors stratified by lowest versus highest quartile of the M-cell signature score; significance was assessed by the log-rank test. **B** M-cell signature score across molecular subtypes in the pre-neoadjuvant chemotherapy MIBC cohort GSE87304; differences across groups were assessed by the Kruskal-Wallis test. **C** H&E and IHC staining with KRT8 and VIM in human sarcomatoid bladder cancer primary tumors (Bca0323, Bca0425 and Bca0826) and patient-derived xenograft tumors in NSG mice. Scale bar: 100 μM.

To determine whether the M-cell transcriptional program identified in normal human urothelium is relevant to bladder cancer, we next evaluated its clinical significance in human bladder tumors. In TCGA-BLCA dataset, tumors with high M-cell signature scores associated with significantly worse overall survival than tumors with low scores (Fig. 7A). In an independent pre-neoadjuvant chemotherapy MIBC cohort (GSE87304), the M-cell signature was significantly enriched in the Claudin-Low subtype, an EMT-enriched tumor state described by Seiler et al. [31] (Fig. 7B). To further validate the clinical relevance in human SARC tumors, we assessed co-expression of KRT8 and vimentin in primary SARC specimens. As expected, colocalization of KRT8 and VIM was observed in primary SARC tumors (Bca0323, Bca0425, Bca0826) and corresponding patient-derived xenografts (Fig. 7C). We also evaluated the expression of additional EMT biomarkers in these tumors. Hybrid expression patterns of EMT-associated markers were observed across the tumor samples (Fig. S2). The Bca0323 tumor exhibited expression of AXL, E-cadherin, and CD44, whereas the Bca0425 tumor showed weak E-cadherin expression but strong expression of ZEB1 and FN1. In contrast, the Bca0826 tumor demonstrated strong expressions of ZEB1, FN1, and CD44. Together, these findings support the biological and clinical relevance of the M-cell transcriptional state in TCGA bladder cancers and human SARC tumors, suggesting a potential role of the M-cells in SARC tumorigenesis.

## Discussion

In this study, we identified a previously unrecognized M-cell population in normal mouse and human urothelium. These cells are defined by a 16-gene M-cell transcriptional signature and characterized by co-expression of epithelial markers (Epcam, Krt8/18) with mesenchymal and stemness genes (Vim, Axl, Twist1, Zeb1, and CD44). Lineage tracing in *Upk2-Cre mT/mG* mice confirmed their urothelial origin and transcriptional M-cell program suggests a potential link between a M-cell state, MB49 tumors, and human SARC tumors.

Single-cell RNA sequencing (scRNA-seq) studies from Yu et al. [19] and others identified multiple epithelial clusters beyond the canonical types, including rare EMT-like or transitional populations. Our analysis uncovered that M-cells (approximately 3% of urothelial cells) correspond to these EMT-like subsets, supported by *Upk2*-driven lineage tracing and co-expression of epithelial genes (Epcam, Krt8, Krt18, Krt19). Seven genes from the Guo EMT signature (Vim, Twist1, Zeb1, Axl, Lix1l, Mmp2, Antxr2) were shared with our 50-gene M-cell program, while the 16-gene subset unique to the urothelium (absent in fibroblasts) defined their specific identity. These M-cells were consistently detected across multiple datasets (GSE129845, GSE164557, GSE240189), highlighting their conservation across species.

The discovery of M-cells also provides insight into urothelial plasticity in tumorigenesis. Variant histology can occur in up to 30% of bladder cancers, with SARC representing one of the most aggressive subtypes [33]. SARC is characterized by frequent *TP53*, *RB1*, and *PIK3CA* alterations and activation of EMT and inflammatory pathways. While SARC is thought to emerge via EMT from preexisting urothelial carcinoma, it is also plausible that these tumors could originate from rare M-cells that acquire oncogenic mutations. This hypothesis is supported by the strong transcriptional similarity (16-gene signature) between M-cells and the MB49 bladder tumor model. MB49 has long been considered fibroblast-like rather than epithelial [41]. Our bulk RNA-seq deconvolution and independent scRNA-seq analyses revealed robust enrichment of the M-cell signature and absence of fibroblast-specific programs. These findings suggest that MB49 tumors are likely to represent a mesenchymal-like urothelial carcinoma rather than a fibroblast-derived sarcoma. Histological evidence of KRT8, AXL, and VIM co-expression and enrichment for EMT signatures further support this possibility. Consistent with this concept, co-expression of VIM and KRT8 was observed in human SARC bladder tumors and corresponding patient-derived xenografts. These dual-positive SARC cells retain both epithelial and mesenchymal features, suggesting a hybrid M-cell state in SARC. CytoTRACE2 analysis showed that M-cells are highly plastic and most enriched in the basal layer. The persistence of the transitional M-cell state in both normal and malignant contexts also raises the possibility that M-cells may represent a preexisting, plastic urothelial population that may contribute to SARC initiation. Future M-cell lineage tracing studies should define whether M-cells act as facultative progenitors activated by oncogenic hits.

The identification of M-cells raises several translational implications. First, M-cells may act as biomarkers of urothelial plasticity associated with more aggressive disease. The M-cells gene and regulon profiles reveal potential therapeutic vulnerabilities in SARC tumors. Specifically, M-cells express AXL, a druggable target implicated in tumor invasion and immune evasion. Bemcentinib (BGB324/R428), a selective AXL inhibitor, has demonstrated anti-tumor activity in advanced non–small cell lung cancer when combined with chemotherapy [43]. In this study, we observed therapeutic activity of Bemcentinib in MB49 tumors, supporting its potential translational application for human SARC (Fig. 6C). Further investigating these agents in patient-derived models could test whether targeting M-cell associated programs improve outcomes in aggressive SARC.

A limitation of our study is that the proposed M-cell plasticity and relationship to MB49 tumors are inferred primarily from transcriptomic similarity based on bulk RNA-seq deconvolution and scRNAseq. A highly plastic M-cell transcriptional program in MB49 tumors does not provide direct evidence of cell-of-origin or functional stem-cell capacity. Future studies, including organoid or sphere formation assays, in vivo tumor serial dilution assays, and lineage-tracing experiments, will be needed to determine whether M-cells possess self-renewal capacity, tumor-initiating capacity, or serve as a true cell of origin for SARC bladder cancer. Another limitation is that this study did not directly evaluate lineage plasticity between conventional urothelial carcinoma and SARC tumors. In SARC patients with conventional urothelial carcinoma or variant histology, the relationship between the SARC component and the conventional urothelial or other variant components was not assessed. Future studies will be needed to define whether SARC components share a common lineage or represent distinct cell-of-origin or tumor evolution processes.

In summary, we identified a rare, transcriptionally distinct M-cell population in the normal mouse and human bladder urothelium. These M-cells retain urothelial identity while exhibiting increased plasticity and enrichment of EMT- and inflammation-related programs. The similarity between M-cells, mouse MB49 tumor, and human SARC bladder tumors supports a potential link between the M-cell state, intrinsic urothelial plasticity, and SARC malignant transformation. The unique M-cell transcriptional program may also reveal new therapeutic targets for SARC bladder cancer.

## Supplementary information

Supplementary figure and Table legends

Figure S1. Colocalization of vimentin with AXL, E-cadherin, and CD44 in human normal bladder tissue.

Figure S2. EMT and stem cell-like marker expression in human sarcomatoid bladder cancer tumors.

Table S1. Differentially expressed genes across broad cell types in the normal mouse bladder.

Table S2. Differentially expressed genes across urothelial cell populations.

Table S3. Shared and cell type-specific genes in M-cells and fibroblasts.

## Data Availability

The bulk RNA-seq data from MB49 tumors used in this study have been previously published [12] by our group and deposited in the GEO database under the accession code GSE200005. Previously published scRNA-seq of normal human bladder samples re-analyzed in this study from Yu et al. [19] is available in GEO as GSE129845. Previously published mouse healthy bladder scRNA-seq data from Cheng et al [5] is available in GEO as GSE164557. Previously published mouse MB49 tumor scRNA-seq data from Zhu et al [42] is available in GEO as GSE240189. TCGA-BLCA RNA-seq and clinical annotations were obtained from TCGA via TCGAbiolinks. Human MIBC microarray data and metadata (including subtype classifications) were obtained from GEO in GSE87304. The mouse normal bladder scRNA-seq and mouse normal bladder bulk RNA-seq data generated in this study are deposited in the GEO database under the accession codes GSE330882 and GSE330902. No original code was produced in this study.
