# Supplementary material for: Single-cell profiling uncovers a rare mesenchymal-like urothelial state associated with sarcomatoid bladder cancer
Source: Cell Death Dis. 2026 Aug 4;17(1):927. doi: 10.1038/s41419-026-09136-8 (PMC13724738; doi:10.1038/s41419-026-09136-8)
Supplement: Supplementary file 1 [file 41419_2026_9136_MOESM1_ESM.docx]

Supplemental Figure Legends

**Fig. S1 Colocalization of vimentin with AXL, E-cadherin, and CD44 in human normal bladder tissue.** Co-immunofluorescence staining of vimentin and AXL or E-cadherin or CD44 in representative human male and female bladder tissues. Scale bar: 100 μM. Yellow arrow: colocalization; White arrow: non-colocalization.

**Fig. S2 EMT and stem cell-like marker expression in human sarcomatoid bladder cancer tumors.** IHC staining with AXL, E-cad, ZEB1, FN1 and CD44 in human sarcomatoid primary tumors (Bca0323, Bca0425 and Bca0826) and patient-derived xenograft tumors in NSG mice. Scale bar: 100 μM.

Supplemental Table Legends

**Table S1. Differentially expressed genes across broad cell types in the normal mouse bladder.** Cell populations include epithelial cells, fibroblasts, stromal cells, endothelial cells, macrophages, monocytes, dendritic cells (DC), neutrophils, T cells, natural killer T cells (NKT), and innate lymphoid cells (ILC). For each gene, the table reports the nominal p-value (p_val), average log_2_ fold change between the indicated cell population and all other cells (avg_log_2_FC), the proportion of cells expressing the gene within the indicated population (pct.1) and among all remaining cells (pct.2), the multiple-testing-adjusted p-value (p_val_adj), the annotated cell population (cluster), and the gene symbol (gene).

**Table S2. Differentially expressed genes across urothelial cell populations.** Differentially expressed genes defined the Basal, Umbrella, Intermediate-1, Intermediate-2, and M-cell populations within the urothelial subset. The table includes average log_2_ fold change, the percentage of cells expressing each gene, and adjusted p-value.

**Table S3. Shared and cell type-specific genes in M-cells and fibroblasts.** Comparison of genes expressed by M-cells and fibroblasts, classified as unique to M-cells, unique to fibroblasts, or shared between both populations. The table indicates the presence of each gene in the two populations and its corresponding overlap category.
